# The effect of gastrostomy tube feeding on growth in children with chronic kidney disease and on dialysis

**DOI:** 10.1007/s00467-024-06277-w

**Published:** 2024-02-13

**Authors:** Abdulelah Alshaiban, Adebola Osuntoki, Shelley Cleghorn, Antonia Loizou, Rukshana Shroff

**Affiliations:** 1 UCL Great Ormond Street Institute of Child Health, University College London, London, WC1N 3JH UK; 2 Department of Pediatrics, College of Medicine, King Saud University, King Saud University Medical City, Riyadh, Saudi Arabia

**Keywords:** Gastrostomy tube feeding, Nutrition, Growth, Children, Chronic kidney disease, Obesity

## Abstract

**Background:**

Gastrostomy tube (GT) feeding is used to promote nutrition and growth in children with chronic kidney disease (CKD). We explored the relationship between gastrostomy feeding and growth parameters in children with CKD, looking specifically at the nutritional composition of feeds.

**Methods:**

Children with CKD stages 3–5 or on dialysis in a tertiary children’s kidney unit were studied. Data on anthropometry, biochemistry, and nutritional composition of feeds were collected from the time of GT insertion for 3 years or until transplantation.

**Results:**

Forty children (18 female) were included. Nineteen children were on peritoneal dialysis, 8 on hemodialysis, and 13 had CKD stages 3–5. The median (interquartile range [IQR]) age at GT insertion was 1.26 (0.61–3.58) years, with 31 (77.5%) under 5 years of age. The median duration of gastrostomy feeding was 5.32 (3.05–6.31) years. None received growth hormone treatment. Children showed a significant increase in weight standard deviation score (SDS) (*p* = 0.0005), weight-for-height SDS (*p* = 0.0007) and body mass index (BMI) SDS (*p* < 0.0001). None of the children developed obesity. Although not statistically significant, the median height-SDS increased into the normal range (from -2.29 to -1.85). Weight-SDS positively correlated with the percentage of energy requirements from feeds (*p* = 0.02), and the BMI-SDS correlated with the percentage of total energy intake as fat (*p* < 0.001).

**Conclusion:**

GT feeding improves weight-SDS and BMI-SDS without leading to obesity. GT feeding improved height-SDS but this did not reach statistical significance, suggesting that factors in addition to nutritional optimization need to be considered for statural growth.

**Graphical abstract:**

**Figure:**
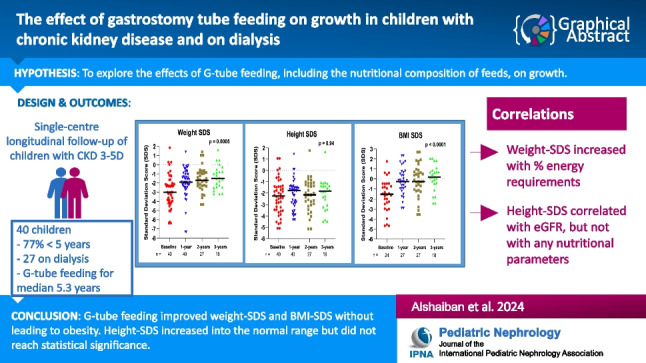
A higher resolution version of the Graphical abstract is available as Supplementary information

**Supplementary Information:**

The online version contains supplementary material available at 10.1007/s00467-024-06277-w.

## Introduction

Poor statural growth is common in children with chronic kidney disease (CKD), manifesting mainly with short stature and poor weight gain [1]. Short stature impairs quality of life, self-esteem and social rehabilitation and is associated with increased mortality [2]. Growth failure becomes more common and more severe as kidney function declines; children with an estimated glomerular filtration rate (eGFR) < 30 ml/min/1.73 m^2^ had a three-fold higher risk of growth failure compared to those with an eGFR ≥ 50 ml/min/1.73 m^2^ [2]. Poor nutrition is a well-described cause of growth failure in CKD; however, even with the institution of an appropriate diet, it is rare for a child with CKD to reach their full height potential. Factors including anemia, metabolic acidosis, bone mineral disease and abnormalities in the somatotropic and gonadotropic hormone axes with growth hormone (GH) resistance also contribute to growth failure [3, 4].

Major efforts have been directed towards improving nutritional intake in children with CKD starting with oral feeding wherever possible. The National Kidney Foundation’s Kidney Disease Outcomes Quality Initiative (KDOQI) and the Pediatric Renal Nutrition Taskforce (PRNT) recommend that in the event of inadequate dietary intake and failure to grow, the diet should be optimized by nutritional supplementation with energy and/or protein [5, 6]. The prescription can be offered initially by mouth, but if there is ongoing failure to thrive, enteral tube feeding, usually via a gastrostomy tube (GT), should be offered [7]. Optimizing nutrition is particularly important in infants and young children when the dependency of growth on nutrition is at its maximum, and if inadequate, can result in the loss of as many as two height-SDS [3].

Enteral feeding is shown to be effective in achieving catch-up growth, as well as improving and maintaining growth in children with CKD [8–10]. Despite its effectiveness, some studies suggest that gastrostomy feeding might improve weight more than height, thereby increasing the risk of obesity in children with CKD, especially for children on peritoneal dialysis [3, 10, 11]. Clearly, the presence or absence of a feeding tube per se cannot affect growth, and the nutritional composition of the feeds delivered through the tube must be carefully considered; this is not described in most studies.

The aim of our study was to explore the relationship between gastrostomy feeding and growth parameters in young children with CKD, looking specifically at the nutritional composition of feeds.

## Methods

### Study population

Children up to the age of 18 years with CKD stages 3–5 and on dialysis who were on enteral feeding through a GT for at least one year in the period between 1999 and 2021 were included. Children were identified from the hospital’s electronic database and dietetic records. Children who used their GT only for fluids and medications, and those in whom anthropometric data at the time of GT insertion were not available were excluded. The study was approved by the local research ethics committee.

### Data collection

Data were collected on patient demographics, age at GT insertion, duration of tube feeds, and significant comorbidities. Data were collected from the point of GT insertion (baseline) for a period of 3 years, or to the time of GT removal, kidney transplantation, or 1 January 2022 (end of study). The nutritional prescription was provided either entirely via the GT (i.e. no oral intake) or partially via the GT to supplement oral nutrition. The exact quantity and nutritional composition of the oral intake showed high intra-individual variability and was not recorded.

Anthropometric data (weight, height and body mass index [BMI]), biochemical markers (including urea, creatinine, urine albumin-creatinine ratio (ACR), glucose, lipid profile and liver enzymes) were collected. Estimated glomerular filtration rate (eGFR) was calculated using the modified Schwartz equation [12]. Dietetic data included details of the feed prescription, total volume of feeds, total calories per day, and nutritional composition of feeds (total carbohydrates, protein, and fat) per day. The percentage of energy requirement (Estimated Average Requirement, (EAR)) from feeds was calculated. The EAR is defined as the average daily requirement that is estimated to meet the needs of 50% of a healthy group of people in the UK, depending on age group and gender. The percentage of the energy from protein, fat and carbohydrates as well as the percentage of the protein requirements from feeds were calculated and expressed as the percentage of Reference Nutrient Intake (RNI). RNI is the amount of a nutrient that is enough to ensure that the needs of 97.5% of a defined cohort are met [13].

Weight standard deviation score (SDS), height-SDS and BMI-SDS were retrieved from growth charts. Weight-for-height SDS was calculated using the World Health Organization (WHO) infant weight-for-length SDS calculator for children below the age of 2, and estimated using the WHO weight-for-height SDS tables for children aged 2 to 5 years [14]. Obesity is defined as a weight-for-height greater than + 3SD, using the WHO child growth standard chart for children 2–5 years of age, and BMI for age greater than + 2SD for children above 5 years of age (equivalent to BMI > 30 kg/m^2^ at 19 years), using the WHO growth reference chart or a country-specific growth chart [15].

Feed prescriptions and EAR were collated from dietetic records. Calculations relating to dietary data include total calories per kilogram, total protein per kilogram, percentage energy requirements (%EAR) from feeds, percentage protein requirements (%RNI) from feeds, and percentages of protein, carbohydrates and fats of total dietary energy, from feeds. Where EAR for energy and RNI for protein were not recorded, it was obtained from the ‘Nutritional Requirements for Children in Health and Disease’ practical guide, produced by the Great Ormond Street Hospital dietetic department using published reference data [13, 16]. In clinical practice, the EAR and RNI for patients’ chronological age is used, with the height age used for children with height < 2nd centile [6].

### Statistical analysis

Assumptions of normality were tested, and data described as median and interquartile range (IQR) for continuous data and as percentages for categorical variables. All anthropometric measures were converted to SDS for comparison across the cohort. Analysis of variance was used to compare the anthropometry, biochemical data and feed composition at baseline and annual follow-up. Chi-square test was used to compare categorical variables. In order to assess the annualized change in anthropometric measures in response to changes in the feed composition, Cox proportional hazards models were fitted to estimate the independent association between time-varying nutritional variables (%EAR for energy, %RNI for protein, % carbohydrate and the % fat of the total dietary energy intake) after adjustment for potential confounders (including the age at start of gastrostomy feeding, prematurity or term birth, CKD or dialysis, and the presence of comorbidities in order to determine the effect of changing dietary prescriptions on anthropometric measures). A two-tailed p value of < 0.05 was considered statistically significant. Data were analyzed using SPSS version 25, and figures were prepared using GraphPad Prism.

## Results

### Demographics

Forty children were included. The median age at the time of GT insertion was 1.26 years (IQR 0.61 to 3.58). Thirty-one (77.5%) children were under 5 years of age at the time of GT insertion. The median duration of gastrostomy feeding was 5.32 (3.05—6.31) years. There are 18 (45%) girls. Seventeen (43%) were White, 14 (35%) Asian, 4 (10%) Black and 5 (12%) of other or mixed ethnicities. Twenty-seven (67%) were on dialysis (19 on peritoneal dialysis and 8 on hemodialysis), 8 (20%) in CKD stages 4—5, and 5 (12%) in stage 3 CKD. The underlying kidney diseases were dysplasia (65%), glomerulonephritis (12.5%), cystic kidney disease (5%) and other or unknown causes (17.5%). Four children had genetic disorders, including Senior-Loken syndrome and Feingold syndrome in one each and mitochondrial disease in two. None of the children in our cohort were on growth hormone.

### Anthropometric data

Anthropometric and biochemical data at baseline and annual follow-up are presented in Table 1.

**Table 1:** Anthropometric and biochemical data of patients at G-tube insertion and one-, two- and three-year follow-up

	At G-tube Insertion (Baseline)	1-year	2-years	3-years	P-value
Number of patients (*n* =)	40	40	27	18	-
Weight SDS	-3.00 (-3.96- -2.11)	-1.91 (-2.45- -1.05)	-1.70 (-2.32- -0.90)	-1.52 (-2.41- -0.78)	0.0005
Height SDS	-2.29 (-3.46- -1.44)	-1.79 (-2.95- -1.52)	-2.15 (-3.02- -1.27)	-1.85 (-3.03- -1.37)	0.94
BMI-for-age SDS	(*n* = 24) -1.56 (-2.49- -0.74)	(*n* = 27) -0.27 (-0.96- 0.49)	(*n* = 27) -0.27 (-1.14- 0.62)	(*n* = 18) 0.17 (-0.40- 0.69)	< 0.0001
Weight-for-height SDS (for patients < 5 years at baseline)	(*n* = 31) -1.17 (-1.98- -0.47)	(*n* = 31) -0.32 (-1.15- 0.61)	(*n* = 24) 0.00 (-1.00- 0.13)	(*n *= 17) 0.00 (0.00- 1.00)	0.0007
Urea * (mmol/L) (Ref. range 2.5 – 6.0)	13.05 (10.35- 18.23)	16.4 (11.75- 20.1)	17 (12.6- 21.45)	16.95 (12.05- 22.2)	0.11
Creatinine * (µmol/L) (Ref. range 15—31)	195 (98.75- 336.5)	349 (146.75- 428.5)	288 (165- 433)	311.5 (201.75- 499.25)	0.009
eGFR * (Ref. range > 90 ml/min)	14.89 (8.47- 29.09)	10.56 (7.49- 20.44)	11.19 (7.63- 20.8)	11.81 (6.73- 16.67)	0.07
ALT * (U/L) (Ref. range 5—45)	26 (20- 32)	28 (22.75- 38.50)	36 (18–82)	31 (20.5- 40.25)	0.22

Values are presented as number (n) and as median with interquartile range (IQR) in parentheses

P-value compares values at baseline, 1, 2, and 3 years, determined by ANOVA multiple analysis of variance. * *n* = 40, 40, 27 and 18 at insertion, 1-year, 2-year and 3-year follow up respectively

*SDS*, standard deviation score; *eGFR*, estimated glomerular filtration rate

**Fig. 1:**
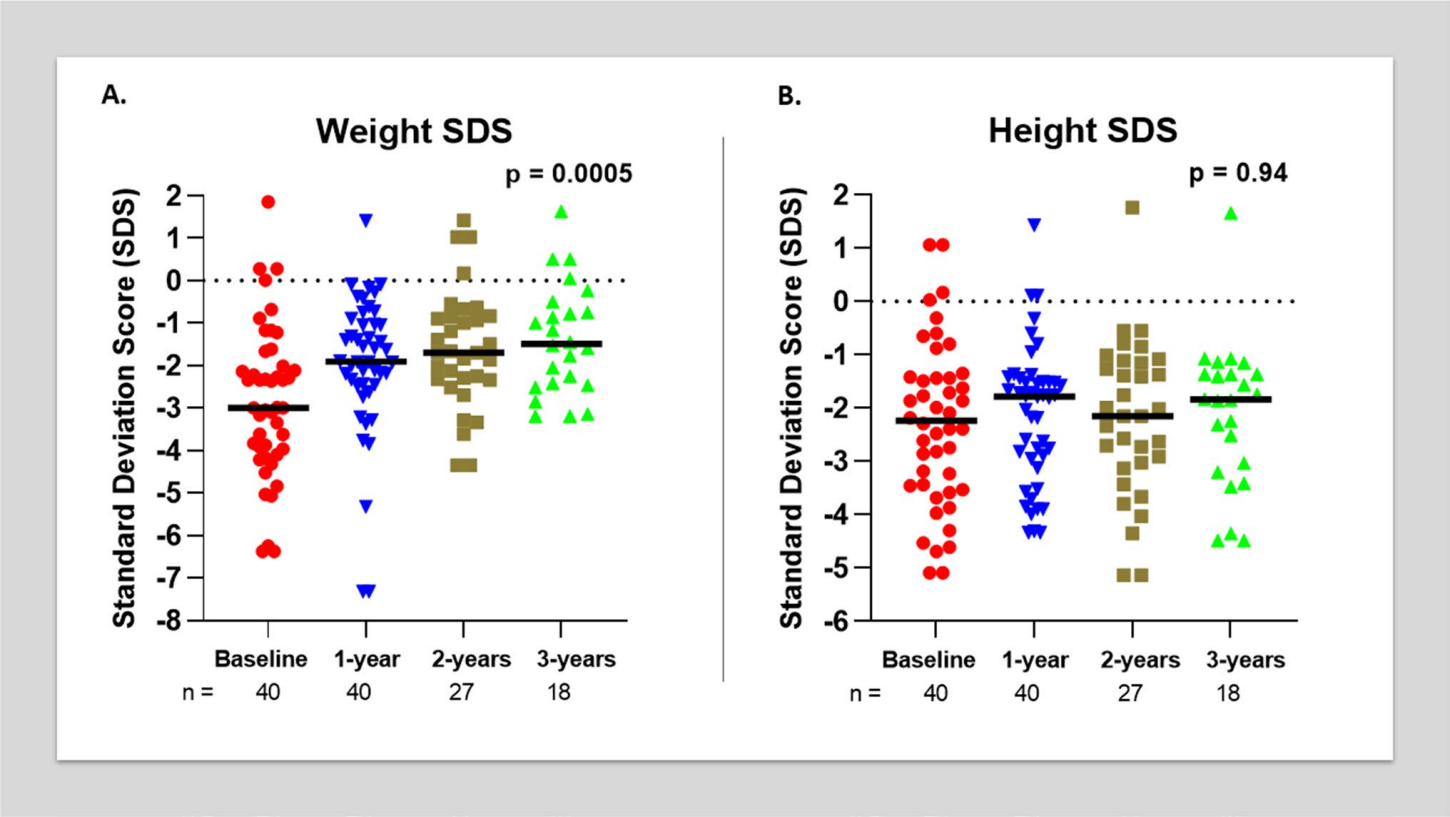
Weight-SDS (**A**) and height-SDS (**B**) of all patients at baseline, one-, two- and three-year follow-up. ‘n’ reprepresents the number of patients, and the black horizontal lines represent the median value for each group

The median weight-SDS and height-SDS at the time of GT insertion were 3.00 and -2.29, respectively (Fig. 1). With the introduction of GT feeding, the weight-SDS improved to -1.52 after 3 years (*p* = 0.0005). The height-SDS increased to -1.85 after 3 years, and although it did not reach statistical significance (*p* = 0.94), it did increase into the normal range. The most significant increase in weight- and height-SDS were seen in the first year of initiatiating GT feeds (-1.91 and -1.79 respectively) (Fig. 1).

At baseline, 77.5% of children had a weight-SDS of less than -2, decreasing to 38.9% by the 3-year follow-up. In contrast, 55% of children had a height-SDS of less than -2 at baseline, decreasing to 44.4% at 3-year follow-up. The weight- and height-SDS and change in SDS over the study period did not differ significantly in children with genetic syndromes (*n* = 4) compared to the overall cohort (*p* = 0.68).

**Fig. 2:**
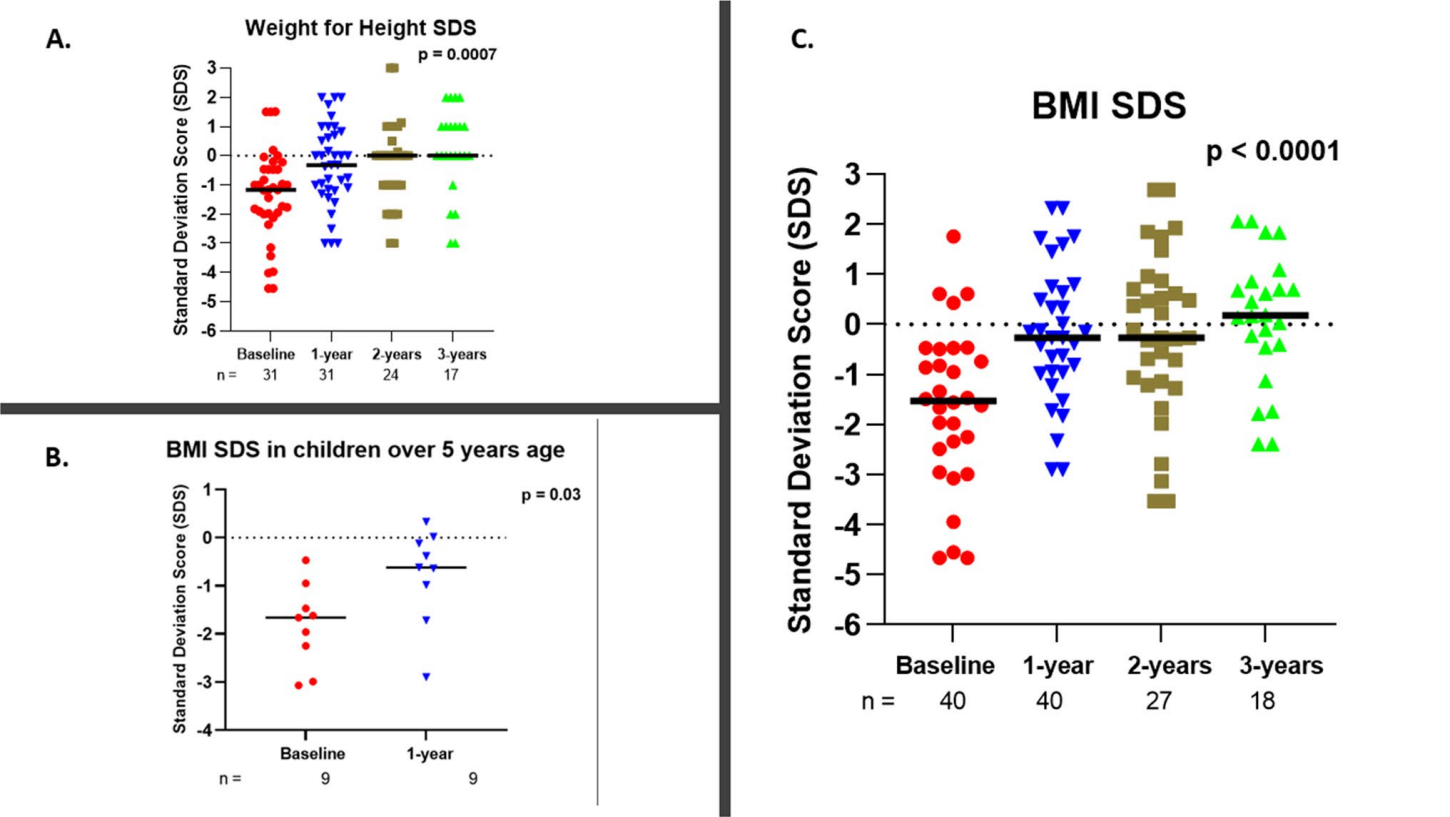
Weight-for-height SDS (**A**) of patients < 5 years old, BMI-SDS of patients > 5 years age (**B**) and BMI-SDS of all patients (**C**) at baseline, one-year, two-year and three-year follow-up. ‘n’ reprepresents the number of patients, and the black horizontal lines represent the median value for each group

Weight-for-height SDS were used to determine obesity in children under the age of five years (*n* = 31), while BMI-for-age SDS were used in children over the age of five (*n* = 9) in accordance with the WHO definition of obesity [14]. There was an increase in weight-for-height SDS from a median of -1.17 at baseline to 0 after 2 years, which was maintained at the 3-year follow-up (Table 1 and Fig. 2). The greatest improvement was seen in the first year after GT insertion (from -1.17 to -0.32). None of the children had a weight-for-height above 3 SD implying that none were obese at any time point in the study. An improvement was also seen in BMI-for-age SDS for children over 5 (*n* = 9) in the first year of follow-up (*p *= 0.03) (Fig. 2). However, due to the small number of children in this group at the 2-year and 3-year follow-up, we did not include these points in our analysis. A statistically significant increase in BMI-SDS (from -1.56 to 0.17, *p* < 0.0001) was seen from baseline to 3 years post-GT insertion (data available in *n* = 24 children). At 3-year follow-up the proportion of children with weight-for-height SDS below -2 decreased from 22.6% at baseline to 5.9%, and those with a BMI-SDS below -2 decreased from 33.3% at baseline to 5.6%.

### Biochemical data

Although serum creatinine levels increased throughout the study (*p* = 0.009), urea levels remained unchanged. Levels of liver transaminases remained within normal limits throughout, indicating that there was no evidence of fatty liver disease. As none of the children in our cohort were obese, by definition, none had non-alcoholic fatty liver disease.

### The nutritional prescription

As the nutritional prescription was tailored to the child’s requirements and adjusted periodically to provide optimal nutrition, there were 36, 37, 24, and 16 different feed prescriptions at baseline, 1-, 2-, and 3-years of follow-up, respectively (Table 2). There were three methods of administering feed prescriptions: full administration via GT, partial administration via GT to supplement oral nutrition, and full oral feeding without the use of a GT. At each data collection point, the majority of patients received partial GT feeds; 72.5%, 82.5%, 63%, and 68.75% at baseline, 1-, 2-, and 3-years of follow-up, respectively (Table 2).

**Table 2:** Dietary data at G-tube insertion and one-, two- and three-year follow-up

	Baseline	1-year	2-years	3-years	P-value
Number of patients in the study (*n* =)	40	40	27	16	-
Fully/Partially Tube fed					-
Partially Fully Orally Unknown	29 (72.5%) 11 (27.5%) 0 0	33 (82.5%) 7 (17.5%) 0 0	17 (63%) 7 (25.9%) 1 (3.7%) 2 (7.4%)	11 (68.75%) 3 (18.75%) 1 (6.25%) 1 (6.25%)	
% Energy requirements (%EAR) from feeds	98.2 (57.2–135.7)	76.8 (61.9–94.4)	69.8 (47.5–98.8)	75.4 (60.2–91.0)	0.14
Total Calories per kg	83 (46–120)	61 (49–75)	55 (39–78)	62 (45–76)	0.07
% Carbohydrates of total dietary energy from feeds	52.6 (44.9–58.0)	48.5 (44.4–56.7)	53.3 (44.4–58.1)	51.3 (44.0–61.1)	0.41
% Fat of total dietary energy from feeds	39.0 (33.3–44.7)	41.3 (34.8–45.1)	38.8 (33.9–44.6)	40.4 (31.5–45.0)	0.89
% Protein of total dietary energy from feeds (PE ratio)	7.8 (6.1–10.4)	8.1 (6.5–10.8)	9.0 (6.0–11.2)	8.5 (6.7–11.0)	0.42
% Protein requirements (%RNI) from feeds	88.3 (62.9–105.1)	105.4 (66.8–141.3)	101.5 (66–142.3)	120.6 (79.7–128)	0.61

Values are presented as number (n) with percentage (%) in parenthesis or as median with interquartile range (IQR) in parenthesis. P-value compares values at baseline, 1, 2, and 3 years, determined by ANOVA multiple analysis of variance

For energy requirements the %EAR at baseline, 1, 2 and 3 years was 98.2%, 76.8%, 69.8% and 75.4%, respectively (Table 2). For protein requirement the %RNI at baseline, 1, 2 and 3 years after GT insertion was 88.3%, 105.4%, 101.5% and 120.6%, respectively (Table 2). Although the median percentage of total energy from feeds decreased during the follow-up period, there was no statistically significant difference (*p* = 0.14). Similarly, the nutritional composition of feeds (% carbohydrates, fats, and proteins of the total dietary energy) showed no differences over the follow-up period.

### Correlation between the nutritional prescription and changes in anthropometry

The weight-SDS increased with an increase in the %EAR from feeds (hazard ratio [HR] 1.81; 95% confidence interval [CI] 1.34 to 4.39; *p* = 0.02) as well as the percentage of total energy intake as fat (HR 2.61; 95% CI 1.72 to 6.09; *p* = 0.005). A linear correlation was seen between BMI-SDS and the percentage of total energy intake as fat (HR 2.33; 95% CI 1.66 to 2.85; *p* < 0.001). No correlations were found between the %EAR from feeds and BMI-SDS (HR 0.87, *p* = 0.22), height-SDS or weight-for-height SDS at any time point.

Height-SDS correlated significantly with eGFR at baseline (*p *= 0.005), and at 1-year follow-up (*p* = 0.04). Similarly, in the weight-for-height SDS correlated positively with eGFR (*p* = 0.03) and inversely with serum urea levels (*p* < 0.001) at baseline. At 1-year follow-up, weight-for-height SDS was inversely associated with urea (*p* < 0.0001) but showed only weak correlation with eGFR (*p* = 0.053). The percentage protein requirement (%RNI) from feed correlated with weight-SDS (*p* = 0.0069, R^2^ = 0.37) and weight-for-height SDS (*p* = 0.03, R^2^ = 0.12) at 12-months, but not at 2- or 3-year follow-up.

At baseline, there are no significant correlations with the percentage protein of total energy intake from feeds (Protein-Energy/PE ratio), but at 1-year, the PE ratio correlated with urea (*p* = 0.05), but not with height-SDS. Furthermore, PE ratio correlated with urea (*p* = 0.053 [borderline]) at 2-year follow-up.

## Discussion

We explored the relationship between enteral feeding and growth parameters in CKD patients and showed that GT feeding leads to a significant improvement in weight-SDS and BMI-SDS. Although the change in height-SDS was not statistically significant, it did show an improvement and increased into the normal range. Other studies have also shown that adequate nutritional supplementation via a tube does lead to an improvement in weight-SDS, BMI-SDS, and height-SDS in CKD children less than 2 years of age [3, 17, 18]. The positive correlation between height-SDS and eGFR suggests that even in very young children statural growth is more affected by kidney function than nutritional intervention alone. Despite early nutritional support, often from birth, and with gastrostomy feeding to overcome issues of CKD-related anorexia, a lower eGFR compromised growth potential. Factors in addition to nutritional optimization by specialist kidney dietitians may need to be considered for those children not demonstrating height catch-up, including administration of recombinant human growth hormone (rhGH). Our results also confirm that despite weight increasing more than height, GT feeding does not lead to obesity.

Short stature and severe growth failure are widely reported in children with CKD [9, 19], especially in those under 2 years of age in whom the height-SDS can decrease to more than -2 SDS without nutritional intervention [3]. The causes of poor nutritional intake in CKD and the adverse effects of this on growth are well-described [10, 11]. Short stature has been linked to higher mortality in the pediatric CKD population [3], and it is thought to be a risk factor for poor transplant prognosis [20]. Many studies on the benefits of enteral feeding are difficult to interpret because of a mixing of ages, CKD stages, and inappropriate or lack of comparator groups. Also, the method of feeds delivery varied amongst children. Ideally, continuous overnight feeding is encouraged with some daytime bolus feeds in children with GT. However, the regimen is adjusted frequently depending on the child’s tolerance and is individualized for each child. Feed adjustments, both in volume and composition, as well as anti-emetic medications are used to minimize vomiting, but some children continue to vomit despite these changes. The loss of nutrition through vomiting is difficult to quantify. In clinical practice the nutritional prescription aims to compensate for the loss of estimated calories from vomiting. In addition, details of the type of nutritional formula administered via the enteral feeding device are rarely provided in the literature. These factors cause difficulties when assessing and comparing outcomes.

Previous studies on the use of enteral feeding in children with CKD have rarely described the effects of the nutritional prescription on growth parameters. Ledermann et. al. showed that the mean percentage energy from enteral feed (as a percentage of the EAR) was 78.9–82.1% for the 0–2 year olds and 51.8–61.3% for the 2–5 year olds). The mean %EAR from food and enteral feed was 96.5–104.2% for 0–2 year olds and 85.6–96.4% for the 2–5 year olds [21]. In comparison, we showed that the median %EAR from GT feeds at baseline, 1, 2 and 3 years was 98.2%, 76.8%, 69.8% and 75.4%, respectively. Simmons et. al. have reported that sufficient caloric supplementation that meets 70% or more of Recommended Dietary Allowance leads to normal linear growth in children on hemodialysis [22]. Sienna et al. have reported that there was significant weight gain but no significant height gain for the duration of GT feeding [23], but details of the nutritional prescription are not provided in this study. Our study identified a significant relationship between the amount of energy and fat provided and the weight-SDS as well as the amount of fat provided and the BMI-SDS. Nutritional requirements are determined based on changes in growth and biochemistry, and feed prescriptions are adjusted accordingly based on feed tolerance. For healthy adults and children over the age of 2, the UK dietary reference values recommend that an average of 50% of total dietary energy intake should come from carbohydrates, and 35% from fats [13]. In our study the fat provision was higher than this (38.8–41.3%). This may be due to feeds needing to be manipulated due to disordered blood biochemistry, or the need to provide concentrated energy in small feed volumes as required for oligo-anuric infants.

Some studies have reported that short stature increases the risk of obesity in children with CKD and could also increase the risk of obesity in adulthood [24]. Although we were unable to investigate other components of the metabolic syndrome, a previous study has confirmed that enteral feeding (providing 58% of energy from carbohydrates and 32% from fats) does not enhance hyperlipidaemia; there were no differences in the levels of total cholesterol, triglycerides, low-density lipoprotein, high-density lipoprotein, apo protein A1, apo A2 or lipoprotein (a) between tube-fed and non-tube-fed children with CKD or on dialysis [25]. The lack of significant height improvement despite regular reviews by a specialist kidney pediatric dietitian calls for consideration of other factors such as dialysis adequacy and the use of growth hormone therapy to improve statural growth.

Despite optimal protein provision in the feed, there was a significant improvement only in weight-SDS but not in height-SDS. Guidelines for protein intake suggest maintaining the intake at a minimum of 100% of the reference values [5, 6], and our study achieved this. A low protein intake has been associated with malnutrition, poor growth and protein-energy wasting all of which are common in the CKD population [6]. Guidelines suggest the percentage of total dietary energy intake from protein (PE ratio) should be kept within the range 7.2–12%, and the PRNT recommends protein intake be targeted at the upper end of this range to achieve optimal growth [6]. In our study the median PE ratio was within range, however, it was closer to the lower end of the range most likely due to controlled protein intake in order to prevent excessive urea levels. Although there is no evidence to suggest what urea levels are optimal in children with CKD, it is suggested that urea levels should be kept below 20 mmol/l [26, 27]. Ledermann et al. showed that the PE ratio in enteral feeds were 5.3–6.4% in the 0–2 year age group, however, the children showed a significant increase in both weight- and height-SDS. It is important to note that the protein intake as a %RNI was 110–117% [21], which may indicate that the total protein intake is as important as the PE ratio. Considering there was a positive correlation between PE ratio and the urea level, care needs to be taken when increasing the PE ratio closer to the upper end of the suggested range.

Our single-centre retrospective study has some limitations. Given that this is a 20-year retrospective review some data were missing, but clearly mentioned for all analyses. Collecting dietary data at the point of GT insertion was challenging. Feed volumes and nutrient prescriptions were changed frequently immediately post-GT insertion if the child was not on nasogastric tube feeding before GT feeding, or if the child’s PD had been held for GT insertion, but these short-term adjustments to the nutritional prescription should not affect the assessment of anthropometric measures that we performed. The percentage of total nutritional intake delivered via the GT varied ranging from children who were entirely tube-fed to children who had some oral intake and used the GT for supplementary feeds, with intraindividual variations. As a result, we categorised children as fully tube fed, partially tube fed, or completely orally fed, and their full feed prescription was used in the results. Nutrient intake from food was not included as this was difficult to quantify. Children on PD receive an additional caloric intake from the dialysis fluid which was not included in our study [23]. Obesity is not clearly defined in children under 2 years of age, as BMI is not recommended in children under 2 [23], hence this was difficult to quantify. Also, data on liver transaminases would have enabled an analysis of the effects of weight gain on the development of non-alcoholic fatty liver disease. Despite the above limitations, our study is one of few papers that have explored the relationship between enteral feeding in children with CKD and growth parameters in detail. Caloric intake and the nutritional composition of GT feeds were explored in depth, which previous studies lack [8, 20]. Areas for future research include exploring the relationship between PE ratio, the percentage of protein requirements from feed and growth in the CKD population.

In conclusion, we have shown that GT feeding improved weight-SDS, weight-for-height SDS and BMI-SDS, while height-SDS increased into the normal range but did not reach statistical significance. Despite careful and frequent monitoring and regular modification of the nutritional composition of feeds, short stature remained an issue in a significant number of children, implying the need to consider interventions such as growth hormone therapy in addition to nutritional optimization.

### Supplementary Information

Below is the link to the electronic supplementary material.

Graphical abstract (PPTX 301 KB)

## Data Availability

The authors confirm that the data supporting the findings of this study are available within the article.
