# Supplementary material for: The effect of gastrostomy tube feeding on growth in children with chronic kidney disease and on dialysis
Source: Pediatr Nephrol. 2024 Feb 13;39(10):3049–56. doi: 10.1007/s00467-024-06277-w (PMC11349843; doi:10.1007/s00467-024-06277-w)
Supplement: Supplementary file 1 — Graphical abstract (PPTX 301 KB) [file 467_2024_6277_MOESM1_ESM.pptx]

## Slide 1
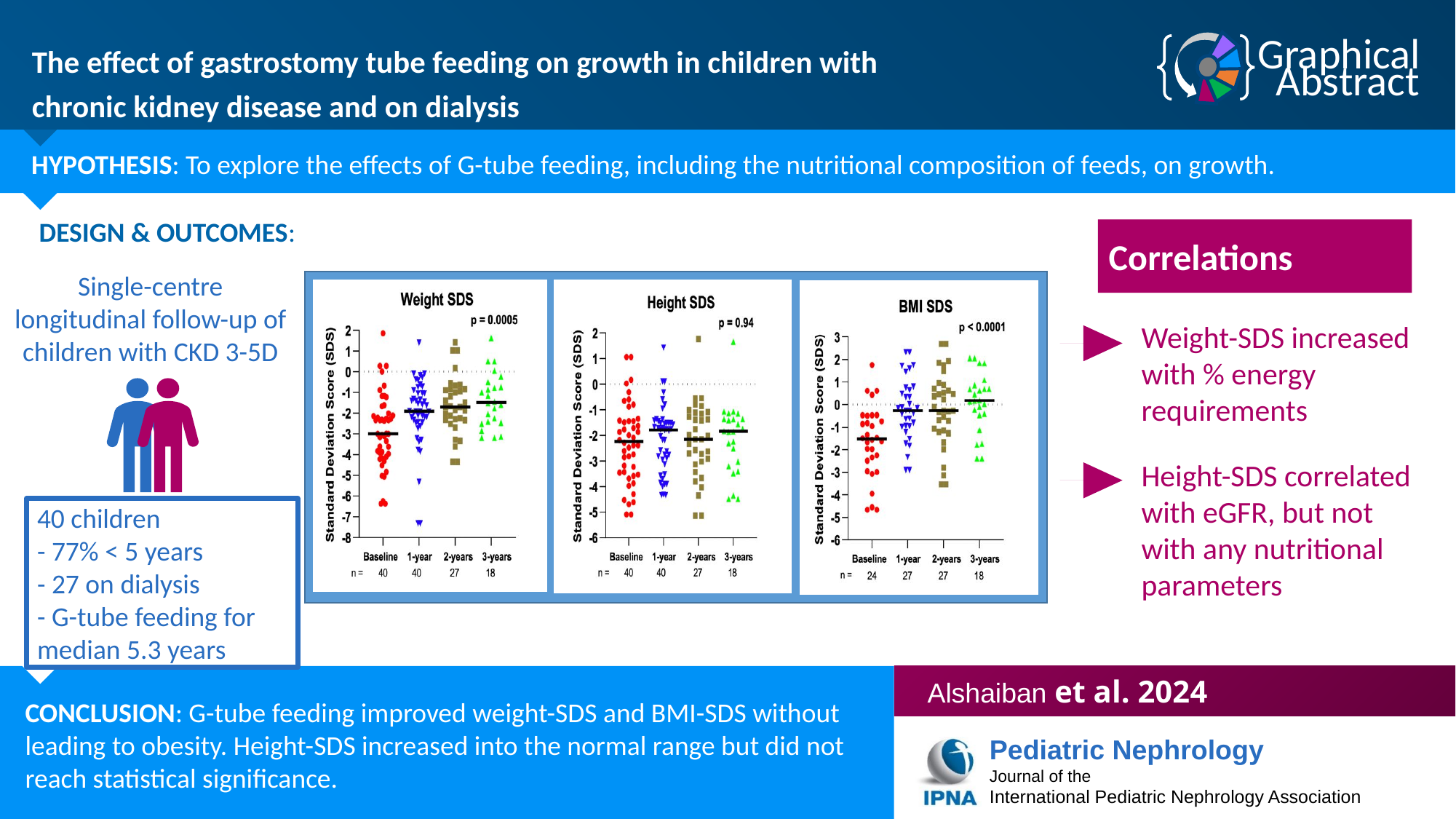

The effect of gastrostomy tube feeding on growth in children with
chronic kidney disease and on dialysis
HYPOTHESIS: To explore the effects of G-tube feeding, including the nutritional composition of feeds, on growth.
DESIGN & OUTCOMES:
Correlations
Single-centre longitudinal follow-up of children with CKD 3-5D
Weight-SDS increased with % energy requirements
Height-SDS correlated with eGFR, but not with any nutritional parameters
40 children
- 77% < 5 years
- 27 on dialysis
- G-tube feeding for median 5.3 years
Alshaiban et al. 2024
CONCLUSION: G-tube feeding improved weight-SDS and BMI-SDS without leading to obesity. Height-SDS increased into the normal range but did not reach statistical significance.
